# Risk of cancer history in cardiovascular disease among individuals with hypertension

**DOI:** 10.1038/s41440-024-01660-4

**Published:** 2024-04-24

**Authors:** Yuta Suzuki, Hidehiro Kaneko, Akira Okada, Katsuhito Fujiu, Norifumi Takeda, Hiroyuki Morita, Akira Nishiyama, Yuichiro Yano, Koichi Node, Hideo Yasunaga, Issei Komuro

**Affiliations:** 1 Department of Cardiovascular Medicine, The University of Tokyo, Tokyo, Japan; 2 Center for Outcomes Research and Economic Evaluation for Health, National Institute of Public Health, Saitama, Japan; 3 The Department of Advanced Cardiology, The University of Tokyo, Tokyo, Japan; 4 Department of Prevention of Diabetes and Lifestyle-Related Diseases, Graduate School of Medicine, The University of Tokyo, Tokyo, Japan; 5 Department of Pharmacology, Faculty of Medicine, Kagawa University, Kagawa, Japan; 6 Department of General Medicine, Juntendo University Faculty of Medicine, Tokyo, Japan; 7 Department of Family Medicine and Community Health, Duke University Durham, Durham, NC USA; 8 Department of Cardiovascular Medicine, Saga University, Saga, Japan; 9 The Department of Clinical Epidemiology and Health Economics, School of Public Health, The University of Tokyo, Tokyo, Japan; 10 International University of Health and Welfare, Tokyo, Japan; 11 Department of Frontier Cardiovascular Science, Graduate School of Medicine, The University of Tokyo, Tokyo, Japan

**Keywords:** Hypertension, Cancer, Onco-cardiology, Epidemiology

## Abstract

Hypertension is the leading risk factor for cardiovascular disease (CVD). Although cancer has recently been increasingly recognized as a novel risk factor for CVD events, little is known about whether co-morbid cancer in individuals with hypertension could further increase the risk of CVD events. We sought to determine the association between the cancer history and the risk of CVD in individuals with hypertension. We retrospectively analyzed a large cohort of 747,620 individuals diagnosed with hypertension from January 2005 through May 2022 using the JMDC Claims Database. Composite CVD events, including myocardial infarction (MI), angina pectoris (AP), stroke, heart failure (HF), and atrial fibrillation (AF), were recorded, and a Cox proportional hazard regression was done to estimate hazard ratios (HR) based on the history of cancer and chemotherapy. 26,531 individuals had a history of cancer. During the mean follow-up period of 1269 ± 962 days, 67,154 composite CVD events were recorded. Compared with individuals without a cancer history, cancer survivors had a higher risk of developing composite CVD events (HR: 1.21, 95% confidence interval [CI]: 1.17–1.26). The HRs (95% CIs) associated with cancer history for MI, AP, stroke, HF, and AF were 1.07 (0.90–1.27), 1.13 (1.06–1.20), 1.14 (1.06–1.24), 1.31 (1.25–1.38), and 1.22 (1.10–1.35), respectively. Lastly, individuals who had received chemotherapy for cancer had a particularly higher risk of developing CVD compared to those who did not undergo chemotherapy. A history of cancer was associated with a greater risk of developing CVD among individuals with hypertension.

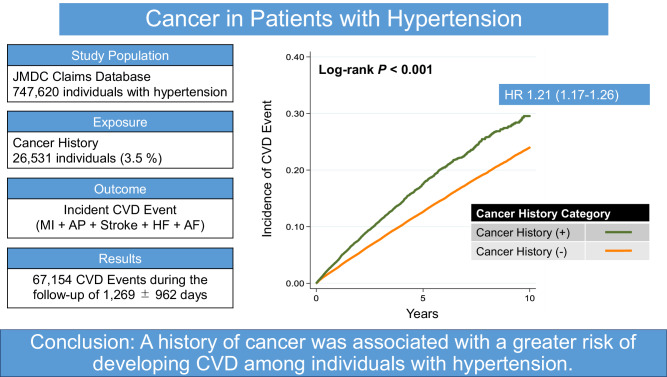

## Introduction

There is growing recognition of the clinical significance of the relationship between hypertension and cancer because the risk of both hypertension and cancer increases with advancing age and some anti-cancer medications can induce hypertension [1]. Recent evidence has suggested that the development of hypertension and certain cancer are interrelated [2–11]. Furthermore, the preceding studies have reported that cancer survivors have a greater risk of subsequent cardiovascular disease (CVD) events, which has led to the emergence of a novel specialization of onco-cardiology [12–14]. For example, previous epidemiological studies demonstrated that hypertension can be associated with a greater risk of some specific cancers [10]. A recent epidemiological study showed that individuals with a history of cancer also had a greater risk of developing hypertension [11]. Further, hypertension was associated with a greater risk of the development of CVD events even in patients with cancer [15]. Considering these potential associations between hypertension and increased cancer risk and the fact that hypertension is the leading cause of CVD, co-morbid cancer in individuals having hypertension could further increase the risk of CVD events. Given this background, it may be also necessary to clarify whether co-morbid cancer in individuals having hypertension leads to a greater risk of CVD events. However, there is a paucity of evidence elaborating the relationship between the presence of cancer and the risk of CVD events in individuals with hypertension. Accordingly, we aimed to investigate the association between cancer and the subsequent risk of CVD among individuals with hypertension using a large-scale epidemiological cohort.

Point of View

- Clinical relevance
  People who have both hypertension and a history of cancer, particularly those receiving active chemotherapy, face a greater risk of developing CVD.
- Future direction
  It is imperative to develop strategies for preventing the onset of CVD events in individuals with hypertension who also have cancer.
- Consideration for the Asian population
  Onco-cardiology is a novel scientific field with significant clinical implications. Our research represents an important epidemiological study in onco-cardiology among Asian populations.

## Methods

### Study population

This retrospective observational cohort study used the JMDC Claims Database (JMDC Inc., Tokyo, Japan) from January 2005 through May 2022 [16–18]. The JMDC Claims Database is a nationwide database that incorporates information on annual health checkups (such as blood pressure [BP] measurement and anthropometric measurements) and individual health insurance records from more than 60 insurers in Japan. The majority of registered individuals were employees who work for relatively large companies in Japan under the coverage of “kempo”, a health insurance system for employees (i.e., working-age population). Because the database includes administrative claims data from both outpatient and inpatient settings, researchers can follow individual patients even if they move to a different hospital during medical treatment. Diagnosis data (confirmed or suspected) in the database are curated according to the International Classification of Diseases, 10th Revision (ICD-10).

For the present study included 999,635 individuals aged 18–74 years with hypertension, more than 1 year after insurance enrollment. We defined hypertension was defined as systolic blood pressure (SBP) ≥ 140 mmHg and diastolic blood pressure ≥ 90 mmHg or requiring the use of the following antihypertensive medications—World Health Organization Anatomical Therapeutic Chemical (WHO-ATC) codes: C02, C03, C04, C07, C08, and C09. Study participants were censored if they reached the age of 75 years or older. Individuals were excluded if they had a history of CVD, including myocardial infarction (MI), angina pectoris (AP), stroke, heart failure (HF), or atrial fibrillation (AF) (*n* = 136,576), history of renal replacement therapy (*n* = 868), or there were missing data regarding cigarette smoking (*n* = 33,157), alcohol consumption (*n* = 54,630), and physical inactivity (*n* = 26,784). Finally, a total of 747,620 patients were included in this study (Supplementary Fig. 1).

### Ethics

This study was approved by the Ethics Committee of the University of Tokyo (approval number: 2018-10862) and conducted in accordance with the principles of the Declaration of Helsinki. Because the JMDC Claims Database included anonymized data, the requirement for informed consent was waived.

### Definition of cancer history

A history of cancer was defined as being confirmed diagnosis with malignant neoplasms (ICD-10 codes: C00-D09) before the index date (i.e., initial health check-up). Additionally, we extracted information regarding the diagnosis of certain cancer types which are reportedly more prevalent in Japanese adults (https://ganjoho.jp/reg_stat/statistics/stat/summary.html). Diagnosis codes of each cancer based on the ICD-10 code are shown in Supplementary Table 1.

### Other variables

Obesity was defined as a body mass index of ≥25 kg/m^2^. Diabetes mellitus was defined as a fasting glucose level of ≥126 mg/dL or the use of blood glucose-lowering medications (including insulin). Dyslipidemia was defined using the following criteria: (1) low-density lipoprotein cholesterol level ≥140 mg/dL; (2) high-density lipoprotein cholesterol level <40 mg/dL; (3) triglyceride level ≥150 mg/dL; or (4) the use of lipid-lowering medications. Information regarding smoking status (current or previous/never) and alcohol consumption (every day or not every day) was obtained by a self-reported questionnaire filled out during the health checkup. Lastly, self-reported physical inactivity was defined as not performing a minimum of 30 min of exercise at least two times a week or not walking for more than 1 h per day.

### Outcomes

The primary outcome was the incidence of composite CVD events, including MI (ICD-10 codes: I21.0–I21.4 and I21.9), AP (ICD-10 codes: I20.0, I20.1, I20.8, and I20.9), stroke (ICD-10 codes: I63.0, I63.1–I63.6, I63.8, I63.9, I60.0–I61.1, I61.3–I61.6, I61.9, I62.9, and G45.9), HF (ICD-10 codes: I50.0, I50.1, I50.9, and I11.0), and AF (ICD-10 codes: I48.0, I48.1, I48.2, I48.3, I48.4, and I48.9) [17, 19]. We also evaluated the individual incidence of MI, AP, stroke, HF, and AF as secondary outcomes. We used confirmed diagnosis records to define each incident of CVD, thereby excluding any suspected diagnoses from the analysis. Study participants were followed until the incidence of outcomes, dropped from insurance coverage, death, or study end date (May 2022).

### Statistical analysis

We used median (25–75th percentiles) or number (percentage) to describe the basic characteristics of study participants stratified by cancer history. For comparing basic characteristics between the two groups, we used the Chi-square test for categorical variables and the Mann-Whitney U test for continuous variables. Additionally, we used the Kaplan–Meier curves with a log-rank test to estimate the incidence of each CVD event according to cancer history. We also constructed a Cox proportional hazard regression model to examine the association between a history of cancer and CVD incidence. Model 1 was an unadjusted model, so we adjusted for age and sex in Model 2; Model 3 was further adjusted for SBP, obesity, diabetes mellitus, dyslipidemia, cigarette smoking, alcohol consumption, and physical inactivity. We also computed the hazard ratios (HRs) for each covariate in the multivariable Cox proportional hazard regression (Model 3).

Furthermore, individuals were categorized into three groups: those without a history of cancer, those with a history of cancer but did not receive active chemotherapy, and those with a history of cancer who received active chemotherapy. Active chemotherapy was defined as the use of antineoplastic agents (WHO-ATC codes: L01) within 3 months before the index date because the Japanese system of universal health insurance allows for a maximum prescription term of 3 months. Using this categorization, we constructed a Cox proportional hazard regression to examine the association between a combination of cancer history and chemotherapy with CVD incidence. We assessed the HRs using Cox proportional hazard regression for composite CVD events among individuals with the five most prevalent cancer types in our dataset.

Additionally, we conducted seven sensitivity analyses. First, we set an induction period of 1 year to exclude individuals with latent CVD. Second, we constructed the Fine-Gray competing risk regression model by accounting for death as a competing risk. Third, we imputed missing data on covariates using multiple imputations by chained equations and 20 iterations, assuming to be missing at random for covariates. Fourth, we performed a subgroup analysis stratified by sex (men, women), age (≥50 years, <50 years), SBP (≥median SBP [140 mmHg], <140 mmHg), and the use of antihypertensive medications (with, without). Fifth, we created a separate cohort after 1:1 matching individuals with a history of cancer and those without a history of cancer according to age (every 5 years), sex, and smoking. Sixth, we excluded cigarette smoking from covariates. Seventh, we repeated the primary analyses among individuals aged ≥60 years. A two-sided *p* value of <0.05 was considered statistically significant. All analyses were performed using the Stata software (version 17; StataCorp LLC, College Station, TX, USA).

## Results

### Basic characteristics

Table 1 summarizes the basic characteristics of the study participants stratified by the presence of cancer history. Among the 747,620 participants with hypertension, 26,531 individuals had a history of cancer. The median age for the entire study cohort was 52 years (25–75th percentiles: 46–59 years); 70.2% (*n* = 524,722) of the subjects were males. The subgroup analysis revealed that individuals with a history of cancer were older adults and less likely to be men compared to those without a history of cancer. Diabetes mellitus was more prevalent in those with a history of cancer, whereas individuals without a history of cancer was more likely to be obese and current smokers.

**Table 1:** Baseline demographic and clinical characteristics of the study participants

	Total	Cancer History (−)	Cancer History (+)	*P* Value
	*n* = 747,620	*n* = 721,089	*n* = 26,531	
Age, years	52 (46–59)	52 (45–59)	59 (52–64)	<0.001
Men, *n* (%)	524,722 (70.2)	509,511 (70.7)	15,211 (57.3)	<0.001
Body Mass Index, kg/m^2^	24.6 (22.2–27.5)	24.6 (22.2–27.6)	23.9 (21.5–26.5)	<0.001
Obesity, *n* (%)	348,056 (46.6)	337,800 (46.8)	10,256 (38.7)	<0.001
Diabetes Mellitus, *n* (%)	92,619 (12.4)	88,607 (12.3)	4,012 (15.1)	<0.001
Dyslipidemia, *n* (%)	449,768 (60.2)	433,948 (60.2)	15,820 (59.6)	0.072
Cigarette Smoking, *n* (%)	204,125 (27.3)	200,033 (27.7)	4,092 (15.4)	<0.001
Alcohol Consumption, *n* (%)	255,504 (34.2)	247,198 (34.3)	8,306 (31.3)	<0.001
Physical Inactivity, *n* (%)	402,973 (53.9)	389,121 (54.0)	13,852 (52.2)	<0.001
Systolic Blood Pressure, mmHg	140 (129–148)	140 (129–148)	138 (126–147)	<0.001
Diastolic Blood Pressure, mmHg	89 (80–95)	89 (80–95)	85 (77–92)	<0.001
Antihypertensive medications, *n* (%)	337,164 (45.1)	321,020 (44.5)	16,144 (60.8)	<0.001
Glucose, mg/dL	98 (90–107)	97 (90–107)	99 (91–109)	<0.001
Low-Density Lipoprotein Cholesterol, mg/dL	125 (105–147)	125 (105–147)	122 (101–143)	<0.001
High-Density Lipoprotein Cholesterol, mg/dL	58 (49–71)	58 (49–71)	62 (51–75)	<0.001
Triglycerides, mg/dL	107 (74–159)	107 (74–159)	102 (72–148)	<0.001

Values are shown as median (25–75th percentiles) unless specified

*P* values were calculated using a Chi-square test for categorical variables and a Mann–Whitney U test for continuous variables

### History of cancer and CVD events in individuals with hypertension

**Fig. 1:**
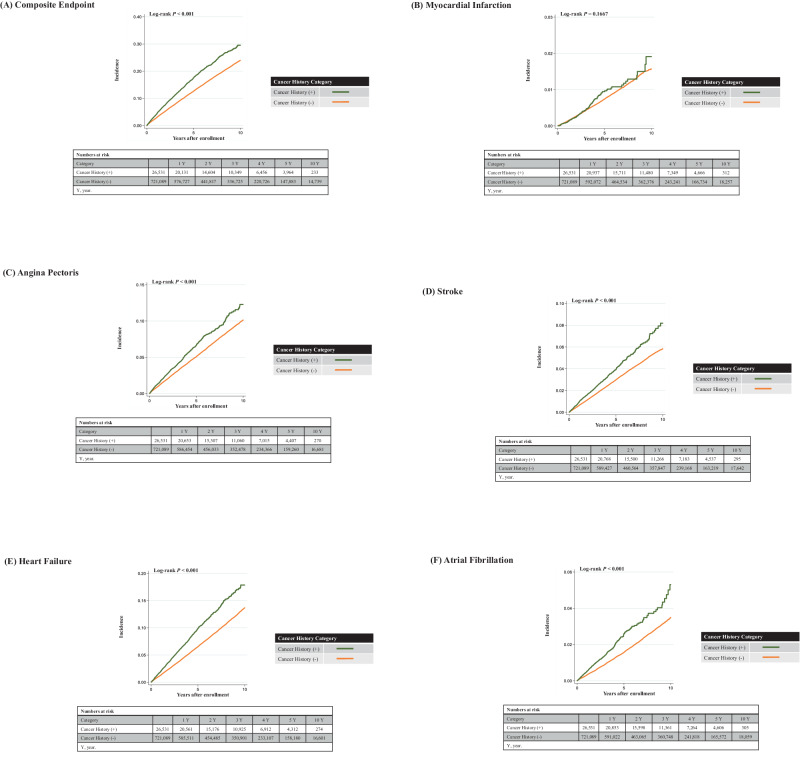
Kaplan–Meier Curves. Kaplan–Meier curves to estimate the cumulative incidence for cardiovascular events ((**A)** composite events, (**B**) myocardial infarction, (**C**) angina pectoris, (**D**) stroke, (**E**) heart failure, and (**F**) atrial fibrillation) according to the presence of cancer history (examined using the log-rank test)

During the mean follow-up period of 1269 ± 962 days, 67,154 composite CVD events (MI: *n* = 3871; AP: *n* = 27,095; stroke: *n* = 15,601; HF: *n* = 34,852; AF: *n* = 8488) were recorded. The log-rank test demonstrated that the incidence of composite CVD event, AP, stroke, HF, and AF was higher in individuals with a history of cancer than in those without (*p* < 0.001) (Fig. 1); however, the incidence of MI was not statistically significant (*p* = 0.1667). Age-sex adjusted survival curves for composite endpoint were also presented in Supplementary Fig. 2. After adjustment for covariates (Model 3), individuals with a history of cancer had a greater risk of composite CVD events than those without (HR: 1.21, 95% confidence interval [95% CI]: 1.17–1.26; AP [HR: 1.13, 95% CI: 1.06–1.20]; stroke [HR: 1.14, 95% CI: 1.06–1.24]; HF [HR: 1.31, 95% CI: 1.25–1.38], and AF [HR: 1.22, 95% CI: 1.10–1.35]) (Table 2). Lastly, the HR for the association between a history of cancer and MI was 1.07 (95% CI: 0.90–1.27). Supplementary Table 2 presents the associations between each covariate and CVD outcome and their HRs.

**Table 2:** Association between cancer history and the risk for cardiovascular disease

	Cancer History (–)	Cancer History (+)
Number	721,089	26,531
Composite Endpoint		
Number of Events	64,264	2890
Incidence (per 10,000 person-years)	273.6 (271.5–275.7)	389.3 (375.4–403.7)
Hazard Ratio (95% Confidence Interval)		
Model 1 (Unadjusted)	1 [Reference]	1.42 (1.37–1.47)
Model 2	1 [Reference]	1.19 (1.15–1.24)
Model 3	1 [Reference]	1.21 (1.17–1.26)
Myocardial Infarction		
Number of Events	3738	133
Incidence (per 10,000 person-years)	14.9 (14.4–15.4)	16.5 (13.9–19.6)
Hazard Ratio (95% Confidence Interval)		
Model 1 (Unadjusted)	1 [Reference]	1.12 (0.94–1.33)
Model 2	1 [Reference]	1.00 (0.84–1.19)
Model 3	1 [Reference]	1.07 (0.90–1.27)
Angina Pectoris		
Number of Events	26,007	1088
Incidence (per 10,000 person-years)	106.4 (105.1–107.7)	139.2 (131.2–147.8)
Hazard Ratio (95% Confidence Interval)		
Model 1 (Unadjusted)	1 [Reference]	1.31 (1.23–1.39)
Model 2	1 [Reference]	1.12 (1.05–1.19)
Model 3	1 [Reference]	1.13 (1.06–1.20)
Stroke		
Number of Events	14,938	663
Incidence (per 10,000 person-years)	60.3 (59.3–61.2)	83.6 (77.5–90.3)
Hazard Ratio (95% Confidence Interval)		
Model 1 (Unadjusted)	1 [Reference]	1.39 (1.28–1.50)
Model 2	1 [Reference]	1.10 (1.01–1.19)
Model 3	1 [Reference]	1.14 (1.06–1.24)
Heart Failure		
Number of Events	33,279	1573
Incidence (per 10,000 person-years)	136.6 (135.1–138.1)	203.1 (193.3–213.4)
Hazard Ratio (95% Confidence Interval)		
Model 1 (Unadjusted)	1 [Reference]	1.50 (1.43–1.58)
Model 2	1 [Reference]	1.28 (1.22–1.35)
Model 3	1 [Reference]	1.31 (1.25–1.38)
Atrial Fibrillation		
Number of Events	8089	399
Incidence (per 10,000 person-years)	32.4 (31.7–33.1)	49.9 (45.3–55.1)
Hazard Ratio (95% Confidence Interval)		
Model 1 (Unadjusted)	1 [Reference]	1.56 (1.41–1.72)
Model 2	1 [Reference]	1.20 (1.09–1.33)
Model 3	1 [Reference]	1.22 (1.10–1.35)

We performed the Cox proportional hazard regression model to examine the association between cancer history and the risk of cardiovascular disease. Model 1 is unadjusted. Model 2 includes adjustment for age and sex. Model 3 includes adjustment for age, sex, systolic blood pressure, obesity, diabetes mellitus, dyslipidemia, cigarette smoking, alcohol consumption, and physical inactivity. The incidence rate was per 10,000 person-years

On analyzing the three groups based on cancer history and the use of antineoplastic agents, cancer patients receiving chemotherapy had the highest risk of each CVD event compared with individuals without a history of cancer, followed by those who did not chemotherapy for cancer. Regarding MI, only cancer patients receiving chemotherapy had a higher risk compared with those without a history of cancer (Table 3). After excluding individuals with multiple cancers history, the five cancer sites with the highest prevalence in men were colorectal (*n* = 3231), prostate (*n* = 2156), stomach (*n* = 1892), renal, pelvic, and ureteral cancer (*n* = 1010), and lung (*n* = 999). On the other hand, breast cancer (*n* = 4468), colorectal cancer (*n* = 1092), thyroid cancer (*n* = 1075), corpus uteri cancer (*n* = 788), and cervix uteri cancer (*n* = 592) were the most prevalent cancer types in women (Supplementary Fig. 3). Men with a history of lung cancer had the highest HR for a composite CVD event compared to those without a history of cancer, whereas females with a history of breast cancer had a higher risk for composite CVD events compared to those without a history of cancer.

**Table 3:** Association of cancer history and chemotherapy with the risk for cardiovascular disease

	Cancer History (–)	Cancer History (+) Chemotherapy (-)	Cancer History (+) Chemotherapy (+)
Number	721,089	25,175	1356
Composite Endpoint			
Number of Events	64,264	2681	209
Incidence (per 10,000 person-years)	273.6 (271.5–275.7)	378.2 (364.2–392.8)	624.1 (545.0–714.7)
Hazard Ratio (95% Confidence Interval)			
Model 1 (Unadjusted)	1 [Reference]	1.38 (1.33–1.43)	2.27 (1.98–2.60)
Model 2	1 [Reference]	1.15 (1.11–1.20)	2.03 (1.77–2.32)
Model 3	1 [Reference]	1.18 (1.13–1.22)	2.09 (1.82–2.39)
Myocardial Infarction			
Number of Events	3738	119	14
Incidence (per 10,000 person-years)	14.9 (14.4–15.4)	15.5 (13.0–18.6)	37.7 (22.3–63.7)
Hazard Ratio (95% Confidence Interval)			
Model 1 (Unadjusted)	1 [Reference]	1.05 (0.88–1.26)	2.58 (1.53–4.36)
Model 2	1 [Reference]	0.94 (0.78–1.13)	2.40 (1.42–4.06)
Model 3	1 [Reference]	1.00 (0.83–1.20)	2.58 (1.53–4.37)
Angina Pectoris			
Number of Events	26,007	1020	68
Incidence (per 10,000 person-years)	106.4 (105.1–107.7)	136.8 (128.7–145.5)	189.6 (149.5–240.5)
Hazard Ratio (95% Confidence Interval)			
Model 1 (Unadjusted)	1 [Reference]	1.29 (1.21–1.37)	1.78 (1.40–2.26)
Model 2	1 [Reference]	1.10 (1.03–1.17)	1.61 (1.27–2.04)
Model 3	1 [Reference]	1.11 (1.04–1.18)	1.62 (1.28–2.06)
Stroke			
Number of Events	14,938	628	35
Incidence (per 10,000 person-years)	60.3 (59.3–61.2)	83.1 (76.8–89.8)	95.7 (68.7–133.3)
Hazard Ratio (95% Confidence Interval)			
Model 1 (Unadjusted)	1 [Reference]	1.38 (1.27–1.49)	1.59 (1.14–2.21)
Model 2	1 [Reference]	1.08 (1.00–1.18)	1.36 (0.98–1.90)
Model 3	1 [Reference]	1.13 (1.04–1.22)	1.46 (1.05–2.04)
Heart Failure			
Number of Events	33,279	1435	138
Incidence (per 10,000 person-years)	136.6 (135.1–138.1)	194.1 (184.3–204.4)	393.4 (333.0–464.9)
Hazard Ratio (95% Confidence Interval)			
Model 1 (Unadjusted)	1 [Reference]	1.43 (1.36–1.51)	2.92 (2.47–3.45)
Model 2	1 [Reference]	1.22 (1.16–1.29)	2.63 (2.23–3.11)
Model 3	1 [Reference]	1.25 (1.19–1.32)	2.75 (2.32–3.25)
Atrial Fibrillation			
Number of Events	8089	375	24
Incidence (per 10000 person-years)	32.4 (31.7–33.1)	49.2 (44.5–54.4)	65.3 (43.8–97.4)
Hazard Ratio (95% Confidence Interval)			
Model 1 (Unadjusted)	1 [Reference]	1.53 (1.38–1.70)	2.04 (1.37–3.05)
Model 2	1 [Reference]	1.18 (1.06–1.31)	1.71 (1.15–2.55)
Model 3	1 [Reference]	1.19 (1.07–1.32)	1.78 (1.19–2.66)

We performed the Cox proportional hazard regression model to examine the association between cancer history and the risk of cardiovascular disease. Individuals were divided into three groups: cancer history absent, cancer history present without chemotherapy, cancer history present with chemotherapy. Model 1 is unadjusted. Model 2 includes adjustment for age and sex. Model 3 includes adjustment for age, sex, systolic blood pressure, obesity, diabetes mellitus, dyslipidemia, cigarette smoking, alcohol consumption, and physical inactivity. The incidence rate was per 10,000 person-years

### Sensitivity analyses

Among the sensitivity analyses, we found that the primary findings were unchanged despite setting an induction period of 1 year (Supplementary Table 3). Second, individuals with a history of cancer had a greater risk of composite CVD events as per the Fine-Gray competing risk regression model (Supplementary Table 4). Third, we repeated the analyses after imputing missing data for fields, such as cigarette smoking, alcohol consumption, and physical inactivity, using multiple imputations with chained equations, and found that the positive association between a history of cancer and CVD events was similar even after multiple imputations (Supplementary Table 5). Fourth, the positive association between a history of cancer and the risk of composite CVD event was consistent across all subgroups (Supplementary Fig. 4). Fifth, 26,531 individuals with a history of cancer were 1:1 matched with 26,531 those without a history of cancer according to age, sex, and smoking, resulting in a matched cohort of 53,062 participants. Even in this population, our main findings did not change (Supplementary Table 6). Sixth, the primary finding was unchanged even after excluding cigarette smoking from covariates (Supplementary Table 7). Seventh, the positive association between a history of cancer and the risk of composite CVD event were also found among individuals aged ≥60 years (Supplementary Table 8).

## Discussion

The present study using a nationwide epidemiological cohort, including 747,620 individuals having hypertension, demonstrated that individuals with a history of cancer had an excess risk of developing various CVD events. The future risk of CVD is further increased in cancer individuals undergoing active chemotherapy.

Onco-cardiology is an emerging scientific and clinical field that has gained prominence in recent years. Once considered “an incurable disease”, there has been immense progress in early diagnostic detection techniques in the field of oncology leading to sustained improvements in prognosis through the use of innovative treatments and the development of supportive care. However, an elevated risk of CVD has been recognized among cancer survivors as a significant epidemiological concern [12–14]. Concurrently, hypertension continues to be a formidable risk factor for the development of various CVD among the general population. Therefore, the coexistence of cancer and hypertension could presumably increase the risk of developing CVD.

The following mechanism could be proposed to explain our findings. It is well-established that cancer survivors are at an excessive risk of CVD, and that several chemotherapeutic agents contribute to this increased risk [14, 20]. On the other hand, medications, such as vascular endothelial growth factor inhibitors, are known to cause severe hypertension which may aggravate the elevated risk of CVD in individuals with hypertension [21–23]. Cancer patients or those undergoing active cancer treatment frequently present with a variety of coexisting conditions, such as chronic kidney disease [24–26]. In such patients, the presence of chronic kidney disease could complicate the antihypertensive treatment and the selection of antihypertensive medications, potentially contributing to inadequate BP management and an increased risk of CVD development. Furthermore, factors such as pain, anxiety, and stress due to coexisting cancer could worsen the hypertensive state subsequently resulting in a greater CVD risk. Additionally, in cancer patients with concomitant hypertension, concerns regarding excessive BP lowering may lead to inadequate antihypertensive treatment and potentially increase the risk of CVD events. The mechanisms underlying non-significant association between a history of cancer and subsequent MI event are unclear, however, there could be a limitation in statistical power due to a small number of events in this study.

The current study has several clinical implications. We demonstrated the clinical relevance of cancer comorbidity in individuals with hypertension. It is noteworthy that the association between cancer history and CVD events assessed using relative risk exceeded the reported association of CVD events with a 5-mmHg increase in SBP among hypertensive patients. Therefore, physicians need to manage hypertensive individuals with the understanding that cancer comorbidity not only adversely affects cancer mortality but also significantly increases the risk of CVD (which is the most important complication in individuals with hypertension). Furthermore, considering that hypertension itself may contribute to increasing cancer risk, greater attention is required toward the early detection of cancer in individuals with hypertension. Since this study used a retrospective observational design, we could not conclude a causal relationship or provide detailed insights into the pathophysiology between cancer history and subsequent CVD events. Future investigations should focus on elucidating the relationship between a history of cancer and the increased risk of CVD in hypertensive individuals, enabling individual assessment of CVD risk and proposing appropriate preventive strategies.

### Limitations

Detailed cancer-related information (e.g., stage of cancer) was unavailable in our dataset. However, the incidence of CVD in our dataset was comparable to that in other epidemiological cohorts in Japan [27, 28]. Furthermore, the specificity of disease diagnoses recorded in the Japanese claims databases is reported to be high [29]. However, there remains uncertainty in the accuracy of recorded diagnoses of cancer or CVD due to a nature of claims databases. The primary findings of our study should be validated using other independent datasets. We performed multivariable analyses and corroborated our primary results with various sensitivity analyses. We also performed a matched analysis to account for large differences in background factors (age, sex, smoking rate) by cancer status to confirm the robustness of the main finding. However, the presence of residual confounding should be recognized as a limitation of our study. Furthermore, it is crucial to acknowledge the absence of data on past-smoking in our database. The JMDC Claims Database predominantly comprises individuals of working age, leading to the inclusion of a comparatively younger demographic in this study. Our findings should be validated using datasets that encompass all age groups, including older adults who are at a higher predisposition for cancer. Although we examined the association between a history of cancer at the initial health checkup and CVD events in this study, the new onset of cancer among individuals without a history of cancer could influence the results. Finally, we could not fully examine the association between specific cancers and CVD incidents due to the small sample size.

### Perspective of Asia

The coexistence of cancer and hypertension is an important clinical challenge in Asian countries, particularly in some developed countries with advanced aging. Through this study, we should recognize that the co-occurrence of cancer in Asian individuals of hypertension also increases the risk of developing subsequent CVD.

## Conclusions

Individuals with hypertension having a history of cancer experience an increased risk of developing CVD, especially those who undergo active chemotherapy. Our results confirm the clinical importance of cancer history in individuals with hypertension from the perspective of CVD prevention.

## Supplementary information

Supplementary Figures

Supplementary Tables

## Data Availability

The JMDC Claims Database is available for purchase from JMDC Inc. (https://www.jmdc.co.jp/en/).
