# Supplementary material for: Risk of cancer history in cardiovascular disease among individuals with hypertension
Source: Hypertens Res. 2024 Apr 24;47(7):1871–80. doi: 10.1038/s41440-024-01660-4 (PMC11224009; doi:10.1038/s41440-024-01660-4)

**Supplementary Figure 1. Flowchart**

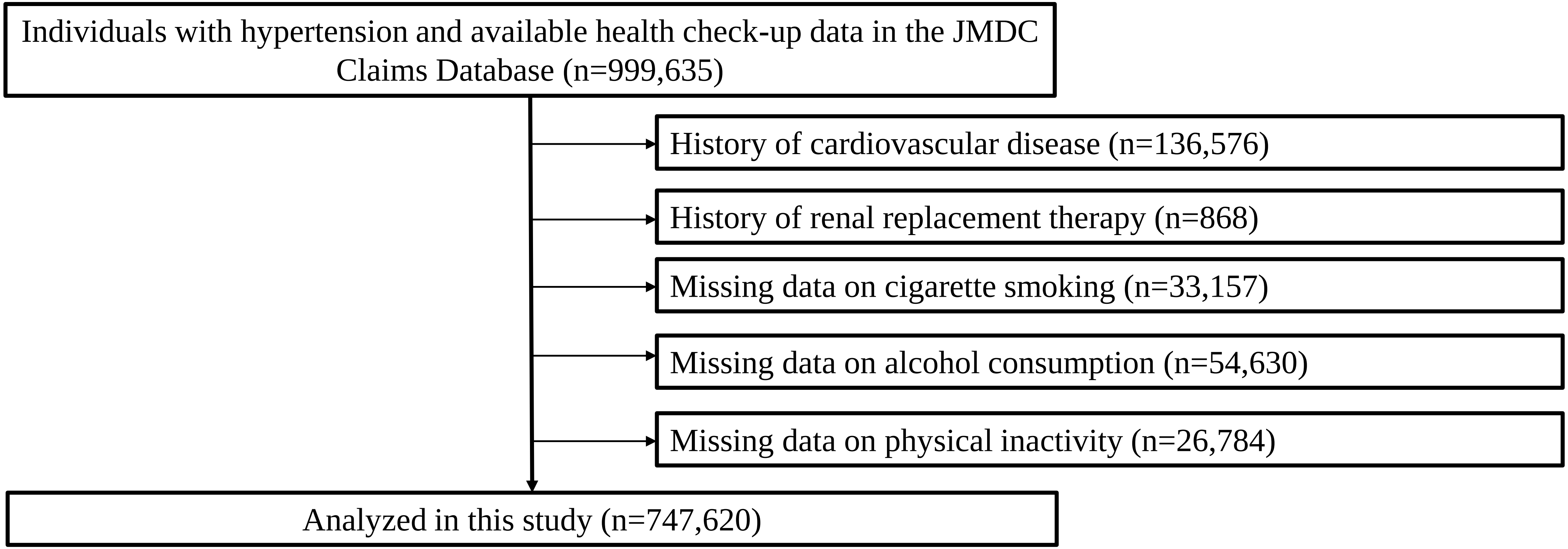

# Supplementary Figure 2. Age-sex Adjusted Survival Curves for Composite Endpoint

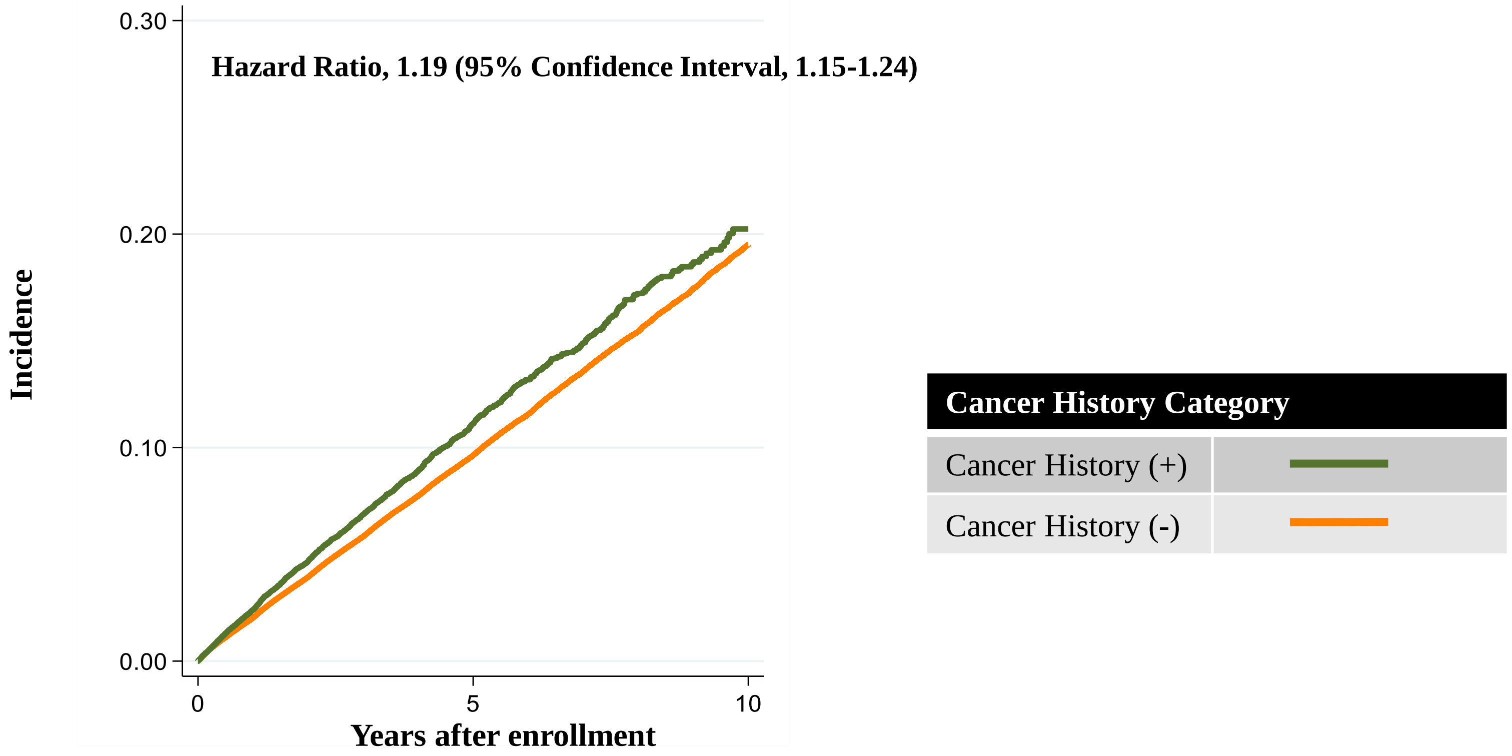

## Supplementary Figure 3. Association between Cancer History with the Highest Number of Patients and the Risk for Composite Cardiovascular Disease

| | No. | No. of Events | Incidence Per 10000 person-years (95% CI) | Model 1 | Model 2 | Model 3 | Hazard Ratio and 95% Confidence Interval in Model 3 |
|---|---|---|---|---|---|---|---|
| **Men** | | | | | | | |
| Cancer History (-) | 509,511 | 48,605 | 280.2 (277.7-282.7) | 1 [Reference] | 1 [Reference] | 1 [Reference] | |
| Colorectal Cancer | 3,231 | 357 | 377.8 (340.6-419.1) | 1.35 (1.21-1.49) | 1.06 (0.96-1.18) | 1.07 (0.97-1.19) | |
| Prostate Cancer | 2,156 | 254 | 454.1 (401.6-513.5) | 1.62 (1.43-1.83) | 1.04 (0.92-1.18) | 1.08 (0.95-1.22) | |
| Stomach Cancer | 1,892 | 223 | 401.8 (352.4-458.2) | 1.43 (1.26-1.63) | 1.05 (0.92-1.20) | 1.11 (0.97-1.27) | |
| Renal Pelvic and Ureteral Cancer | 1,010 | 126 | 437.9 (367.7-521.4) | 1.56 (1.31-1.86) | 1.16 (0.98-1.39) | 1.16 (0.97-1.38) | |
| Lung Cancer | 999 | 151 | 544.5 (464.2-638.6) | 1.94 (1.65-2.28) | 1.49 (1.27-1.74) | 1.54 (1.31-1.81) | |
| **Women** | | | | | | | |
| Cancer History (-) | 211,578 | 15,659 | 254.9 (250.9-258.9) | 1 [Reference] | 1 [Reference] | 1 [Reference] | |
| Breast Cancer | 4,468 | 390 | 322.4 (292.0-356.1) | 1.26 (1.14-1.40) | 1.15 (1.04-1.27) | 1.16 (1.05-1.28) | |
| Colorectal Cancer | 1,092 | 82 | 289.8 (233.4-359.8) | 1.13 (0.91-1.41) | 0.95 (0.77-1.19) | 0.96 (0.77-1.19) | |
| Thyroid Cancer | 1,075 | 92 | 302.3 (246.4-370.9) | 1.19 (0.97-1.46) | 1.11 (0.90-1.36) | 1.12 (0.92-1.38) | |
| Corpus Uteri Cancer | 788 | 72 | 333.6 (264.8-420.3) | 1.31 (1.04-1.65) | 1.24 (0.98-1.56) | 1.21 (0.96-1.53) | |
| Cervix Uteri Cancer | 592 | 48 | 279.9 (210.9-371.4) | 1.10 (0.83-1.46) | 1.12 (0.85-1.49) | 1.14 (0.86-1.51) | |

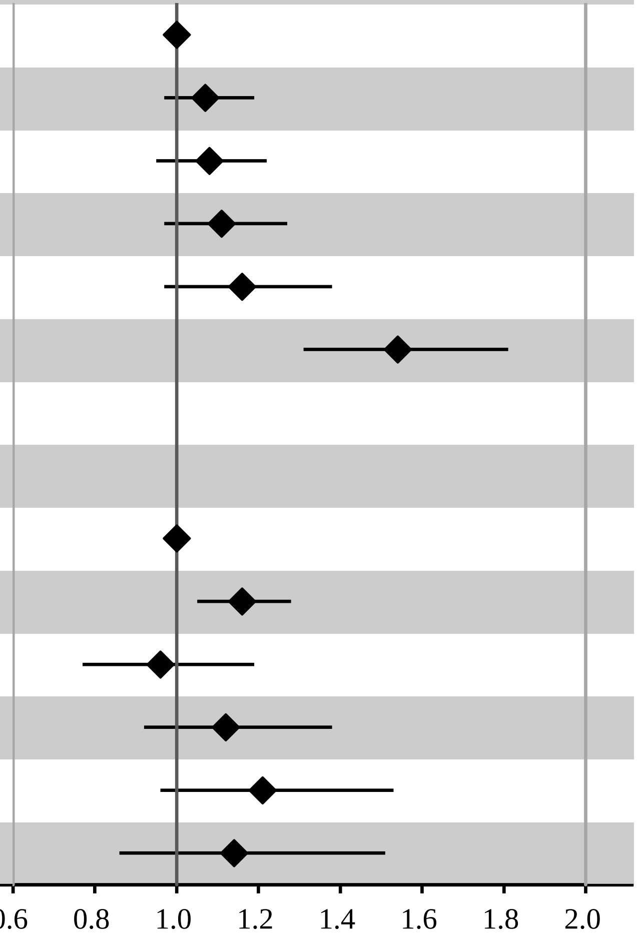

# Supplementary Figure 4. Subgroup Analysis of Association between Cancer History and the Risk for Composite Cardiovascular Disease

| | Number | Number of Events | Incidence Per 10000 person-years (95% CI) | Model 1 | Model 2 | Model 3 | Forest Plot |
|---|---|---|---|---|---|---|---|
| **Men** | | | | | | | |
| Cancer History (-) | 509,511 | 48,605 | 280.2 (277.7-282.7) | 1 [Reference] | 1 [Reference] | 1 [Reference] | |
| Cancer History (+) | 15,211 | 1,884 | 431.9 (412.8-451.8) | 1.54 (1.47-1.61) | 1.20 (1.14-1.26) | 1.22 (1.17-1.28) | |
| **Women** | | | | | | | |
| Cancer History (-) | 211,578 | 15,659 | 254.9 (250.9-258.9) | 1 [Reference] | 1 [Reference] | 1 [Reference] | |
| Cancer History (+) | 11,320 | 1,006 | 328.6 (308.9-349.5) | 1.29 (1.21-1.37) | 1.17 (1.10-1.25) | 1.18 (1.11-1.26) | |
| **Age ≥ 50 years** | | | | | | | |
| Cancer History (-) | 435,860 | 43,714 | 329.3 (326.2-332.4) | 1 [Reference] | 1 [Reference] | 1 [Reference] | |
| Cancer History (+) | 22,180 | 2,444 | 412.3 (396.3-429.0) | 1.25 (1.20-1.30) | 1.16 (1.11-1.21) | 1.18 (1.13-1.23) | |
| **Age < 50 years** | | | | | | | |
| Cancer History (-) | 285,229 | 20,550 | 201.2 (198.5-204.0) | 1 [Reference] | 1 [Reference] | 1 [Reference] | |
| Cancer History (+) | 4,351 | 446 | 298.0 (271.6-327.0) | 1.49 (1.35-1.63) | 1.40 (1.28-1.54) | 1.45 (1.32-1.59) | |

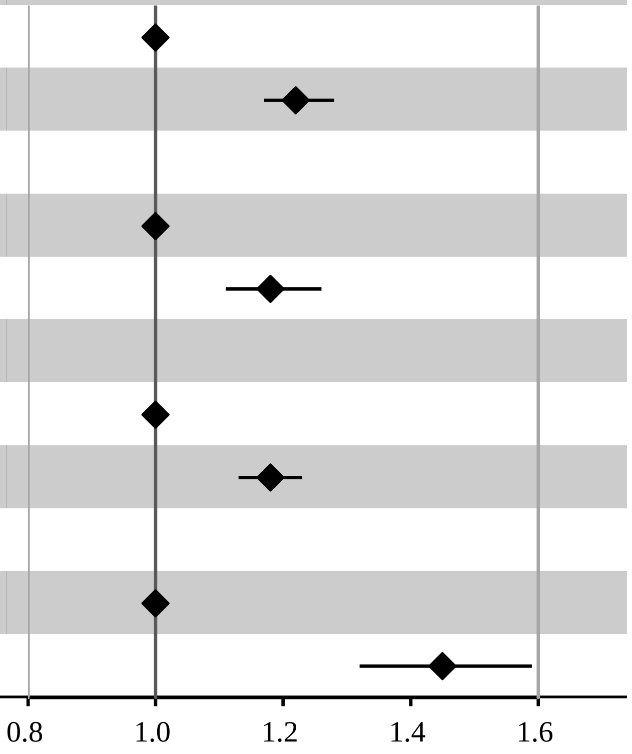

# Supplementary Figure 4. Subgroup Analysis of Association between Cancer History and the Risk for Composite Cardiovascular Disease

| | Number | Number of Events | Incidence Per 10000 person-years (95% CI) | Model 1 | Model 2 | Model 3 |
|---|---|---|---|---|---|---|
| **SBP ≥ 140 mmHg** | | | | | | |
| Cancer History (-) | 374,379 | 32,928 | 271.7 (268.8-274.7) | 1 [Reference] | 1 [Reference] | 1 [Reference] |
| Cancer History (+) | 12,154 | 1,282 | 379.2 (359.0-400.6) | 1.39 (1.32-1.47) | 1.16 (1.10-1.23) | 1.20 (1.14-1.27) |
| **SBP < 140 mmHg** | | | | | | |
| Cancer History (-) | 346,710 | 31,336 | 275.5 (272.5-278.6) | 1 [Reference] | 1 [Reference] | 1 [Reference] |
| Cancer History (+) | 14,377 | 1,608 | 397.7 (378.7-417.6) | 1.44 (1.37-1.52) | 1.21 (1.15-1.27) | 1.22 (1.16-1.28) |
| **With Antihypertensive Medication** | | | | | | |
| Cancer History (-) | 321,020 | 33,569 | 329.8 (326.3-333.3) | 1 [Reference] | 1 [Reference] | 1 [Reference] |
| Cancer History (+) | 16,144 | 1,888 | 426.2 (407.4-445.9) | 1.29 (1.23-1.35) | 1.16 (1.11-1.22) | 1.17 (1.12-1.23) |
| **Without Antihypertensive Medication** | | | | | | |
| Cancer History (-) | 400,069 | 30,695 | 230.6 (228.0-233.2) | 1 [Reference] | 1 [Reference] | 1 [Reference] |
| Cancer History (+) | 10,387 | 1,002 | 334.7 (314.6-356.0) | 1.45 (1.36-1.55) | 1.21 (1.13-1.29) | 1.26 (1.18-1.34) |

**Hazard Ratio and 95% Confidence Interval in Model 3**

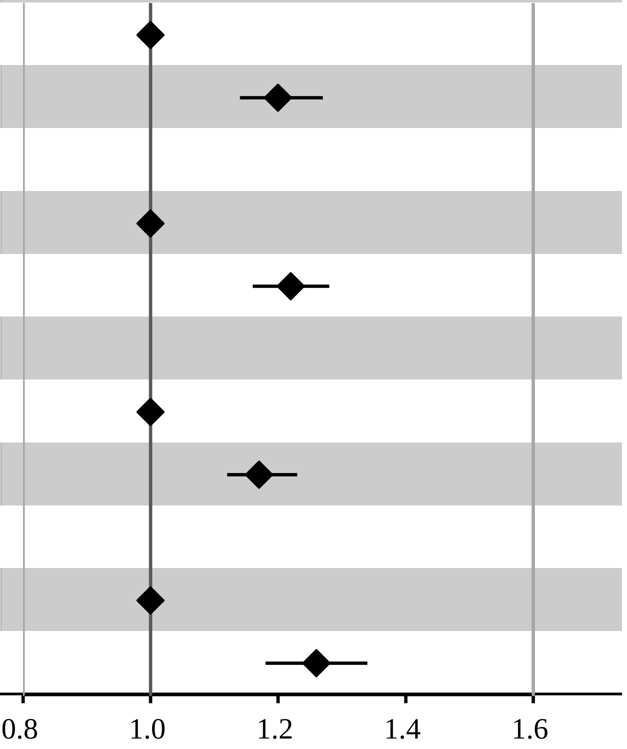

Supplement: Supplementary file 1 — Supplementary Figures [file 41440_2024_1660_MOESM1_ESM.pdf]
