# Supplementary material for: Risk of cancer history in cardiovascular disease among individuals with hypertension
Source: Hypertens Res. 2024 Apr 24;47(7):1871–80. doi: 10.1038/s41440-024-01660-4 (PMC11224009; doi:10.1038/s41440-024-01660-4)
Supplement: Supplementary file 2 — Supplementary Tables [file 41440_2024_1660_MOESM2_ESM.docx]

**Supplementary Table 1. ICD-10 Codes of Cancer Diagnosis**

| Cancer type | ICD-10 codes |
| --- | --- |
| Malignant neoplasms (Total cancer) | C00-D09 |
| Head and neck | C00-C14, C32 |
| Esophagus | C15 |
| Thyroid | C73 |
| Lung | C34 |
| Stomach | C16 |
| Colorectal | C18-C20 |
| Liver | C22 |
| Gallbladder | C23 |
| Pancreas | C25 |
| Kidney | C64 |
| Renal pelvis and ureteral | C65-C68 |
| Leukemia | C91-C93 |
| Non-Hodgkin lymphoma | C85 |
| Malignant melanoma | C43 |
| Malignant neoplasm without specification of site | C80 |
| Prostate (Men) | C61 |
| Breast (Women) | C50 |
| Cervix Uteri (Women) | C53 |
| Corpus Uteri (Women) | C54 |

ICD-10, International Classification of Diseases, 10th revision.

| **Supplementary Table 2 Hazard ratios for Cardiovascular Disease** | | | | | | |
| --- | --- | --- | --- | --- | --- | --- |
|  | Composite | MI | AP | Stroke | HF | AF |
| Cancer History (+) | 1.21 (1.17-1.26) | 1.07 (0.90-1.27) | 1.13 (1.06-1.20) | 1.14 (1.06-1.24) | 1.31 (1.25-1.38) | 1.22 (1.10-1.35) |
| Age (per 5 years increase) | 1.21 (1.20-1.21) | 1.26 (1.23-1.28) | 1.19 (1.18-1.20) | 1.29 (1.28-1.30) | 1.20 (1.19-1.20) | 1.39 (1.37-1.41) |
| Men | 1.13 (1.11-1.15) | 2.65 (2.39-2.93) | 1.17 (1.14-1.21) | 1.15 (1.11-1.20) | 1.16 (1.13-1.19) | 2.09 (1.97-2.23) |
| SBP (per 5 mmHg increase) | 1.02 (1.02-1.02) | 1.03 (1.02-1.04) | 1.00 (1.00-1.01) | 1.05 (1.04-1.05) | 1.03 (1.03-1.03) | 1.00 (1.00-1.01) |
| Obesity | 1.15 (1.13-1.17) | 1.16 (1.08-1.24) | 1.12 (1.09-1.15) | 1.04 (1.01-1.08) | 1.22 (1.20-1.25) | 1.31 (1.26-1.37) |
| Diabetes Mellitus | 1.23 (1.20-1.25) | 1.65 (1.53-1.78) | 1.32 (1.28-1.36) | 1.18 (1.13-1.24) | 1.24 (1.20-1.28) | 1.09 (1.03-1.16) |
| Dyslipidemia | 1.07 (1.05-1.08) | 1.70 (1.58-1.83) | 1.19 (1.16-1.22) | 1.04 (1.00-1.07) | 1.04 (1.02-1.06) | 0.84 (0.81-0.88) |
| Cigarette Smoking | 1.15 (1.13-1.17) | 1.92 (1.79-2.05) | 1.16 (1.13-1.19) | 1.31 (1.26-1.36) | 1.16 (1.14-1.19) | 1.10 (1.05-1.15) |
| Alcohol Consumption | 0.91 (0.90-0.93) | 0.51 (0.47-0.55) | 0.87 (0.85-0.89) | 0.90 (0.87-0.94) | 0.88 (0.86-0.90) | 1.12 (1.07-1.17) |
| Physical Inactivity | 1.04 (1.02-1.05) | 1.09 (1.02-1.16) | 1.06 (1.03-1.08) | 1.05 (1.02-1.08) | 1.03 (1.01-1.06) | 1.01 (0.97-1.06) |
| We performed the Cox proportional hazard regression model including age, sex, systolic blood pressure, obesity, diabetes mellitus, dyslipidemia, cigarette smoking, alcohol consumption, and physical inactivity. Hazard ratios (95% confidence interval) are presented. | | | | | | |

| **Supplementary Table 3. Association between Cancer History and the Risk for Cardiovascular Disease (Induction Period of One Year)** | | |
| --- | --- | --- |
|  | Cancer History (-) | Cancer History (+) |
| Number | 577,106 | 20,145 |
| **Composite Endpoint** |  |  |
| Number of Events | 46,022 | 1,936 |
| Incidence (per 10000 person-years) | 270.2 (267.8-272.7) | 378.8 (362.3-396.1) |
| Hazard Ratio (95% Confidence Interval) |  |  |
| Model 1 (Unadjusted) | 1 [Reference] | 1.40 (1.34-1.47) |
| Model 2 | 1 [Reference] | 1.19 (1.14-1.25) |
| Model 3 | 1 [Reference] | 1.21 (1.16-1.27) |
| **Myocardial Infarction** |  |  |
| Number of Events | 2,769 | 103 |
| Incidence (per 10000 person-years) | 15.3 (14.8-15.9) | 18.7 (15.5-22.7) |
| Hazard Ratio (95% Confidence Interval) |  |  |
| Model 1 (Unadjusted) | 1 [Reference] | 1.23 (1.01-1.50) |
| Model 2 | 1 [Reference] | 1.13 (0.93-1.38) |
| Model 3 | 1 [Reference] | 1.21 (0.99-1.47) |
| **Angina Pectoris** |  |  |
| Number of Events | 18,246 | 712 |
| Incidence (per 10000 person-years) | 103.3 (101.8-104.8) | 133.0 (123.6-143.1) |
| Hazard Ratio (95% Confidence Interval) |  |  |
| Model 1 (Unadjusted) | 1 [Reference] | 1.29 (1.20-1.39) |
| Model 2 | 1 [Reference] | 1.12 (1.04-1.21) |
| Model 3 | 1 [Reference] | 1.14 (1.05-1.23) |
| **Stroke** |  |  |
| Number of Events | 10,646 | 441 |
| Incidence (per 10000 person-years) | 59.5 (58.4-60.7) | 81.3 (74.0-89.2) |
| Hazard Ratio (95% Confidence Interval) |  |  |
| Model 1 (Unadjusted) | 1 [Reference] | 1.37 (1.24-1.50) |
| Model 2 | 1 [Reference] | 1.10 (0.99-1.21) |
| Model 3 | 1 [Reference] | 1.14 (1.04-1.26) |
| **Heart Failure** |  |  |
| Number of Events | 23,991 | 1,074 |
| Incidence (per 10000 person-years) | 136.3 (134.6-138.1) | 202.4 (190.7-214.9) |
| Hazard Ratio (95% Confidence Interval) |  |  |
| Model 1 (Unadjusted) | 1 [Reference] | 1.50 (1.41-1.59) |
| Model 2 | 1 [Reference] | 1.30 (1.22-1.38) |
| Model 3 | 1 [Reference] | 1.33 (1.25-1.41) |
| **Atrial Fibrillation** |  |  |
| Number of Events | 5,748 | 267 |
| Incidence (per 10000 person-years) | 31.9 (31.1-32.8) | 48.8 (43.3-55.1) |
| Hazard Ratio (95% Confidence Interval) |  |  |
| Model 1 (Unadjusted) | 1 [Reference] | 1.55 (1.37-1.75) |
| Model 2 | 1 [Reference] | 1.22 (1.08-1.38) |
| Model 3 | 1 [Reference] | 1.23 (1.09-1.40) |
| We performed the Cox proportional hazard regression model to examine the association between cancer history and the risk of cardiovascular disease. Model 1 is unadjusted. Model 2 includes adjustment for age and sex. Model 3 includes adjustment for age, sex, systolic blood pressure, obesity, diabetes mellitus, dyslipidemia, cigarette smoking, alcohol consumption, and physical inactivity. The incidence rate was per 10,000 person-years. | | |

| **Supplementary Table 4. Association between Cancer History and the Risk for Cardiovascular Disease (Competing Risk Model)** | | |
| --- | --- | --- |
|  | Cancer History (-) | Cancer History (+) |
| Number | 721,089 | 26,531 |
| **Composite Endpoint** |  |  |
| Number of Events | 64,264 | 2,890 |
| Incidence (per 10000 person-years) | 273.6 (271.5-275.7) | 389.3 (375.4-403.7) |
| Hazard Ratio (95% Confidence Interval) |  |  |
| Model 1 (Unadjusted) | 1 [Reference] | 1.41 (1.36-1.46) |
| Model 2 | 1 [Reference] | 1.18 (1.14-1.23) |
| Model 3 | 1 [Reference] | 1.20 (1.16-1.25) |
| **Myocardial Infarction** |  |  |
| Number of Events | 3,738 | 133 |
| Incidence (per 10000 person-years) | 14.9 (14.4-15.4) | 16.5 (13.9-19.6) |
| Hazard Ratio (95% Confidence Interval) |  |  |
| Model 1 (Unadjusted) | 1 [Reference] | 1.11 (0.93-1.32) |
| Model 2 | 1 [Reference] | 0.99 (0.83-1.18) |
| Model 3 | 1 [Reference] | 1.06 (0.89-1.26) |
| **Angina Pectoris** |  |  |
| Number of Events | 26,007 | 1,088 |
| Incidence (per 10000 person-years) | 106.4 (105.1-107.7) | 139.2 (131.2-147.8) |
| Hazard Ratio (95% Confidence Interval) |  |  |
| Model 1 (Unadjusted) | 1 [Reference] | 1.30 (1.22-1.38) |
| Model 2 | 1 [Reference] | 1.11 (1.04-1.18) |
| Model 3 | 1 [Reference] | 1.12 (1.05-1.19) |
| **Stroke** |  |  |
| Number of Events | 14,938 | 663 |
| Incidence (per 10000 person-years) | 60.3 (59.3-61.2) | 83.6 (77.5-90.3) |
| Hazard Ratio (95% Confidence Interval) |  |  |
| Model 1 (Unadjusted) | 1 [Reference] | 1.38 (1.27-1.49) |
| Model 2 | 1 [Reference] | 1.09 (1.00-1.17) |
| Model 3 | 1 [Reference] | 1.13 (1.05-1.22) |
| **Heart Failure** |  |  |
| Number of Events | 33,279 | 1,573 |
| Incidence (per 10000 person-years) | 136.6 (135.1-138.1) | 203.1 (193.3-213.4) |
| Hazard Ratio (95% Confidence Interval) |  |  |
| Model 1 (Unadjusted) | 1 [Reference] | 1.49 (1.41-1.57) |
| Model 2 | 1 [Reference] | 1.27 (1.21-1.34) |
| Model 3 | 1 [Reference] | 1.30 (1.24-1.37) |
| **Atrial Fibrillation** |  |  |
| Number of Events | 8,089 | 399 |
| Incidence (per 10000 person-years) | 32.4 (31.7-33.1) | 49.9 (45.3-55.1) |
| Hazard Ratio (95% Confidence Interval) |  |  |
| Model 1 (Unadjusted) | 1 [Reference] | 1.54 (1.39-1.70) |
| Model 2 | 1 [Reference] | 1.19 (1.07-1.32) |
| Model 3 | 1 [Reference] | 1.20 (1.09-1.33) |
| We performed the Fine-Gray competing risk regression model to examine the association between cancer history and the risk of cardiovascular disease. Model 1 is unadjusted. Model 2 includes adjustment for age and sex. Model 3 includes adjustment for age, sex, systolic blood pressure, obesity, diabetes mellitus, dyslipidemia, cigarette smoking, alcohol consumption, and physical inactivity. The incidence rate was per 10,000 person-years. | | |

| **Supplementary Table 5. Association between Cancer History and the Risk for Cardiovascular Disease (Multiple Imputation)** | | |
| --- | --- | --- |
|  | Cancer History (-) | Cancer History (+) |
| Number | 831,929 | 30,262 |
| **Composite Endpoint** |  |  |
| Number of Events | 76,747 | 3,397 |
| Incidence (per 10000 person-years) | 271.9 (269.9-273.8) | 391.9 (379.0-405.3) |
| Hazard Ratio (95% Confidence Interval) |  |  |
| Model 1 (Unadjusted) | 1 [Reference] | 1.44 (1.39-1.49) |
| Model 2 | 1 [Reference] | 1.20 (1.16-1.24) |
| Model 3 | 1 [Reference] | 1.22 (1.18-1.26) |
| **Myocardial Infarction** |  |  |
| Number of Events | 4,453 | 154 |
| Incidence (per 10000 person-years) | 14.7 (14.3-15.2) | 16.3 (14.0-19.1) |
| Hazard Ratio (95% Confidence Interval) |  |  |
| Model 1 (Unadjusted) | 1 [Reference] | 1.12 (0.96-1.32) |
| Model 2 | 1 [Reference] | 1.00 (0.85-1.17) |
| Model 3 | 1 [Reference] | 1.07 (0.91-1.25) |
| **Angina Pectoris** |  |  |
| Number of Events | 31,293 | 1,272 |
| Incidence (per 10000 person-years) | 106.4 (105.2-107.6) | 139.1 (131.7-147.0) |
| Hazard Ratio (95% Confidence Interval) |  |  |
| Model 1 (Unadjusted) | 1 [Reference] | 1.31 (1.24-1.38) |
| Model 2 | 1 [Reference] | 1.11 (1.05-1.18) |
| Model 3 | 1 [Reference] | 1.13 (1.06-1.19) |
| **Stroke** |  |  |
| Number of Events | 17,786 | 778 |
| Incidence (per 10000 person-years) | 59.6 (58.7-60.5) | 83.9 (78.2-90.0) |
| Hazard Ratio (95% Confidence Interval) |  |  |
| Model 1 (Unadjusted) | 1 [Reference] | 1.41 (1.31-1.51) |
| Model 2 | 1 [Reference] | 1.10 (1.02-1.18) |
| Model 3 | 1 [Reference] | 1.15 (1.07-1.23) |
| **Heart Failure** |  |  |
| Number of Events | 39,872 | 1,848 |
| Incidence (per 10000 person-years) | 136.0 (134.7-137.4) | 204.2 (195.1-213.7) |
| Hazard Ratio (95% Confidence Interval) |  |  |
| Model 1 (Unadjusted) | 1 [Reference] | 1.52 (1.45-1.59) |
| Model 2 | 1 [Reference] | 1.29 (1.23-1.35) |
| Model 3 | 1 [Reference] | 1.32 (1.26-1.38) |
| **Atrial Fibrillation** |  |  |
| Number of Events | 9,535 | 456 |
| Incidence (per 10000 person-years) | 31.7 (31.1-32.3) | 48.8 (44.5-53.4) |
| Hazard Ratio (95% Confidence Interval) |  |  |
| Model 1 (Unadjusted) | 1 [Reference] | 1.56 (1.42-1.71) |
| Model 2 | 1 [Reference] | 1.19 (1.08-1.31) |
| Model 3 | 1 [Reference] | 1.20 (1.09-1.32) |
| We performed the Cox proportional hazard regression model to examine the association between cancer history and the risk of cardiovascular disease. Model 1 is unadjusted. Model 2 includes adjustment for age and sex. Model 3 includes adjustment for age, sex, systolic blood pressure, obesity, diabetes mellitus, dyslipidemia, cigarette smoking, alcohol consumption, and physical inactivity. The incidence rate was per 10,000 person-years. | | |

| **Supplementary Table 6. Association between Cancer History and the Risk for Cardiovascular Disease (Matching for Age, Sex, and Smoking)** | | |
| --- | --- | --- |
|  | Cancer History (-) | Cancer History (+) |
| Number | 26,531 | 26,531 |
| **Composite Endpoint** |  |  |
| Number of Events | 2,524 | 2,890 |
| Incidence (per 10000 person-years) | 326.1 (313.7-339.1) | 389.3 (375.4-403.7) |
| Hazard Ratio (95% Confidence Interval) |  |  |
| Model 1 (Unadjusted) | 1 [Reference] | 1.19 (1.13-1.26) |
| Model 2 | 1 [Reference] | 1.19 (1.13-1.25) |
| Model 3 | 1 [Reference] | 1.18 (1.12-1.25) |
| **Myocardial Infarction** |  |  |
| Number of Events | 115 | 133 |
| Incidence (per 10000 person-years) | 13.9 (11.5-16.6) | 16.5 (13.9-19.6) |
| Hazard Ratio (95% Confidence Interval) |  |  |
| Model 1 (Unadjusted) | 1 [Reference] | 1.20 (0.93-1.53) |
| Model 2 | 1 [Reference] | 1.19 (0.93-1.53) |
| Model 3 | 1 [Reference] | 1.17 (0.91-1.51) |
| **Angina Pectoris** |  |  |
| Number of Events | 1,030 | 1,088 |
| Incidence (per 10000 person-years) | 127.5 (119.9-135.5) | 139.2 (131.2-147.8) |
| Hazard Ratio (95% Confidence Interval) |  |  |
| Model 1 (Unadjusted) | 1 [Reference] | 1.09 (1.00-1.19) |
| Model 2 | 1 [Reference] | 1.09 (1.00-1.18) |
| Model 3 | 1 [Reference] | 1.08 (0.99-1.18) |
| **Stroke** |  |  |
| Number of Events | 610 | 663 |
| Incidence (per 10000 person-years) | 74.5 (68.8-80.7) | 83.6 (77.5-90.3) |
| Hazard Ratio (95% Confidence Interval) |  |  |
| Model 1 (Unadjusted) | 1 [Reference] | 1.12 (1.00-1.25) |
| Model 2 | 1 [Reference] | 1.12 (1.00-1.25) |
| Model 3 | 1 [Reference] | 1.12 (1.00-1.25) |
| **Heart Failure** |  |  |
| Number of Events | 1,250 | 1,573 |
| Incidence (per 10000 person-years) | 155.2 (146.8-164.0) | 203.1 (193.3-213.4) |
| Hazard Ratio (95% Confidence Interval) |  |  |
| Model 1 (Unadjusted) | 1 [Reference] | 1.31 (1.22-1.41) |
| Model 2 | 1 [Reference] | 1.31 (1.21-1.41) |
| Model 3 | 1 [Reference] | 1.31 (1.21-1.41) |
| **Atrial Fibrillation** |  |  |
| Number of Events | 340 | 399 |
| Incidence (per 10000 person-years) | 41.2 (37.0-45.8) | 49.9 (45.3-55.1) |
| Hazard Ratio (95% Confidence Interval) |  |  |
| Model 1 (Unadjusted) | 1 [Reference] | 1.22 (1.05-1.41) |
| Model 2 | 1 [Reference] | 1.21 (1.04-1.40) |
| Model 3 | 1 [Reference] | 1.21 (1.05-1.40) |
| We performed the Cox proportional hazard regression model to examine the association between cancer history and the risk of cardiovascular disease. Model 1 is unadjusted. Model 2 includes adjustment for age and sex. Model 3 includes adjustment for age, sex, systolic blood pressure, obesity, diabetes mellitus, dyslipidemia, cigarette smoking, alcohol consumption, and physical inactivity. The incidence rate was per 10,000 person-years. | | |

| **Supplementary Table 7. Association between Cancer History and the Risk for Cardiovascular Disease (Excluding Smoking from Covariates)** | | |
| --- | --- | --- |
|  | Cancer History (-) | Cancer History (+) |
| Number | 721,089 | 26,531 |
| **Composite Endpoint** |  |  |
| Number of Events | 64,264 | 2,890 |
| Incidence (per 10000 person-years) | 273.6 (271.5-275.7) | 389.3 (375.4-403.7) |
| Hazard Ratio (95% Confidence Interval) |  |  |
| Model 3 | 1 [Reference] | 1.20 (1.16-1.25) |
| **Myocardial Infarction** |  |  |
| Number of Events | 3,738 | 133 |
| Incidence (per 10000 person-years) | 14.9 (14.4-15.4) | 16.5 (13.9-19.6) |
| Hazard Ratio (95% Confidence Interval) |  |  |
| Model 3 | 1 [Reference] | 1.01 (0.85-1.20) |
| **Angina Pectoris** |  |  |
| Number of Events | 26,007 | 1,088 |
| Incidence (per 10000 person-years) | 106.4 (105.1-107.7) | 139.2 (131.2-147.8) |
| Hazard Ratio (95% Confidence Interval) |  |  |
| Model 3 | 1 [Reference] | 1.12 (1.05-1.19) |
| **Stroke** |  |  |
| Number of Events | 14,938 | 663 |
| Incidence (per 10000 person-years) | 60.3 (59.3-61.2) | 83.6 (77.5-90.3) |
| Hazard Ratio (95% Confidence Interval) |  |  |
| Model 3 | 1 [Reference] | 1.12 (1.03-1.21) |
| **Heart Failure** |  |  |
| Number of Events | 33,279 | 1,573 |
| Incidence (per 10000 person-years) | 136.6 (135.1-138.1) | 203.1 (193.3-213.4) |
| Hazard Ratio (95% Confidence Interval) |  |  |
| Model 3 | 1 [Reference] | 1.30 (1.23-1.37) |
| **Atrial Fibrillation** |  |  |
| Number of Events | 8,089 | 399 |
| Incidence (per 10000 person-years) | 32.4 (31.7-33.1) | 49.9 (45.3-55.1) |
| Hazard Ratio (95% Confidence Interval) |  |  |
| Model 3 | 1 [Reference] | 1.21 (1.09-1.34) |
| We performed the Cox proportional hazard regression model to examine the association between cancer history and the risk of cardiovascular disease. Model 3 includes adjustment for age, sex, systolic blood pressure, obesity, diabetes mellitus, dyslipidemia, alcohol consumption, and physical inactivity. The incidence rate was per 10,000 person-years. | | |

| **Supplementary Table 8. Association between Cancer History and the Risk for Cardiovascular Disease (Aged ≥ 60 years)** | | |
| --- | --- | --- |
|  | Cancer History (-) | Cancer History (+) |
| Number | 161,232 | 12,131 |
| **Composite Endpoint** |  |  |
| Number of Events | 16,332 | 1,381 |
| Incidence (per 10000 person-years) | 409.0 (402.7-415.3) | 492.1 (466.8-518.7) |
| Hazard Ratio (95% Confidence Interval) |  |  |
| Model 1 (Unadjusted) | 1 [Reference] | 1.20 (1.14-1.27) |
| Model 2 | 1 [Reference] | 1.15 (1.08-1.21) |
| Model 3 | 1 [Reference] | 1.16 (1.09-1.22) |
| **Myocardial Infarction** |  |  |
| Number of Events | 913 | 64 |
| Incidence (per 10000 person-years) | 21.1 (19.8-22.6) | 20.9 (16.3-26.6) |
| Hazard Ratio (95% Confidence Interval) |  |  |
| Model 1 (Unadjusted) | 1 [Reference] | 0.99 (0.77-1.27) |
| Model 2 | 1 [Reference] | 0.95 (0.73-1.22) |
| Model 3 | 1 [Reference] | 0.98 (0.76-1.26) |
| **Angina Pectoris** |  |  |
| Number of Events | 6,260 | 508 |
| Incidence (per 10000 person-years) | 149.1 (145.4-152.8) | 171.0 (156.8-186.5) |
| Hazard Ratio (95% Confidence Interval) |  |  |
| Model 1 (Unadjusted) | 1 [Reference] | 1.15 (1.05-1.25) |
| Model 2 | 1 [Reference] | 1.10 (1.01-1.21) |
| Model 3 | 1 [Reference] | 1.11 (1.01-1.21) |
| **Stroke** |  |  |
| Number of Events | 4,265 | 344 |
| Incidence (per 10000 person-years) | 100.4 (97.5-103.5) | 114.2 (102.8-127.0) |
| Hazard Ratio (95% Confidence Interval) |  |  |
| Model 1 (Unadjusted) | 1 [Reference] | 1.14 (1.02-1.27) |
| Model 2 | 1 [Reference] | 1.07 (0.96-1.20) |
| Model 3 | 1 [Reference] | 1.10 (0.99-1.23) |
| **Heart Failure** |  |  |
| Number of Events | 8,278 | 740 |
| Incidence (per 10000 person-years) | 198.3 (194.1-202.6) | 251.4 (234.0-270.2) |
| Hazard Ratio (95% Confidence Interval) |  |  |
| Model 1 (Unadjusted) | 1 [Reference] | 1.27 (1.18-1.37) |
| Model 2 | 1 [Reference] | 1.21 (1.12-1.30) |
| Model 3 | 1 [Reference] | 1.23 (1.14-1.32) |
| **Atrial Fibrillation** |  |  |
| Number of Events | 2,619 | 222 |
| Incidence (per 10000 person-years) | 61.1 (58.8-63.5) | 73.1 (64.1-83.4) |
| Hazard Ratio (95% Confidence Interval) |  |  |
| Model 1 (Unadjusted) | 1 [Reference] | 1.20 (1.05-1.38) |
| Model 2 | 1 [Reference] | 1.13 (0.98-1.29) |
| Model 3 | 1 [Reference] | 1.13 (0.99-1.30) |
| We performed the Cox proportional hazard regression model to examine the association between cancer history and the risk of cardiovascular disease. Model 1 is unadjusted. Model 2 includes adjustment for age and sex. Model 3 includes adjustment for age, sex, systolic blood pressure, obesity, diabetes mellitus, dyslipidemia, cigarette smoking, alcohol consumption, and physical inactivity. The incidence rate was per 10,000 person-years. | | |
